# Identification and functional characterization of the sex-determining gene *doublesex* in the sawfly, *Athalia rosae* (Hymenoptera: Tenthredinidae)

**DOI:** 10.1007/s13355-017-0502-3

**Published:** 2017-06-03

**Authors:** Shotaro Mine, Megumi Sumitani, Fugaku Aoki, Masatsugu Hatakeyama, Masataka G. Suzuki

**Affiliations:** 1 Department of Integrated Biosciences, Graduate School of Frontier Sciences, The University of Tokyo, 5-1-5 Kashiwanoha, Kashiwa-shi, Chiba, 277-8562 Japan; 2 Genetically Modified Organism Research Center, National Institute of Agrobiological Sciences, Owashi, Tsukuba, 305-8634 Japan; 3 Division of Insect Sciences, National Institute of Agrobiological Sciences, Owashi, Tsukuba, 305-8634 Japan

**Keywords:** Hymenoptera, *Athalia rosae*, Sex determination, *Doublesex*, Genitalia

## Abstract

Sexual fate of the sawfly, *Athalia rosae* (Hymenoptera: Tenthredinidae) is determined by the complementary sex determination (CSD) mechanism as is the case in honeybees. However, to date, genes involved in sex determination have not been identified in this species. In this study, we attempted to identify orthologs of *complementary sex*-*determiner* (*csd*), *feminizer* (*fem*), and *doublesex* (*dsx*) from the *A*. *rosae* genome, all of which are crucial components of the sex determination cascade in the honeybee. As a result, we identified a sawfly ortholog of *dsx* (designated as *Ardsx*). Rapid amplification of cDNA ends (RACE) using total RNA extracted from male and female larvae identified three male-specific variants and three female-specific variants. Comparison between the full-length *Ardsx* cDNAs and the genomic sequence revealed that exon 5 was differentially spliced between the male- and female-specific variants. RT-PCR analysis demonstrated that *Ardsx* pre-mRNA was spliced alternatively in a sex-dependent manner at almost all the developmental stages. RNAi-mediated knockdown of *Ardsx* in males caused severe defects in the reproductive organs and, notably, induced development of the ovipository apparatus containing the dorsal pair of blades and the sheath. These males also showed abnormalities in testes and seminal vesicles and lacked mature sperm. The present study provides the first direct evidence that *dsx* is essential for sexual development in hymenopteran species.

## Introduction

In several hymenopteran insects, sexual fate is determined by the complementary sex determination (CSD) mechanism, in which heterozygosity at a single locus (the CSD locus) determines femaleness in diploid individuals, while haploid individuals are hemizygous for the CSD locus and thus develop into males (Whiting 1933). The CSD locus was first molecularly identified in the honeybee *Apis mellifera* and found to be a homolog of *transformer* (*tra*) (Beye et al. 2003). The *tra* gene is known to be a conserved upstream component of the insect sex determination cascade and induces female development by regulating sex-specific alternative splicing of downstream targets such as *doublesex* (*dsx*) (Gempe and Beye 2011; Hoshijima et al. 1991). *A*. *mellifera* has two copies of *tra* homologs (Hasselmann et al. 2008). One copy is named *complementary sex*-*determiner* (*csd*) that is the primary signal for femaleness. It activates the other copy, named *feminizer* (*fem*), which is more conserved and retains the ancestral function of regulating sex-specific alternative splicing *dsx*. The *csd* was considered to have arisen from duplication of the *fem* gene (Schmieder et al. 2012).

Orthologs of *csd* and *fem* have been identified not only in honeybee species but also in bumblebees and ants (Privman et al. 2013; Schmieder et al. 2012). Evolutional analyses demonstrate that the duplication of *fem* that yielded *csd* occurred before the divergence of *Aculeata* species (bees and ants), and also provide evidence that these two genes evolved concertedly through gene conversion. On the basis of these findings, it is supposed that *csd* likely represents the molecular basis for the CSD mechanism in the *Aculeata* species and, possibly, in the entire Hymenoptera order.

**Fig. 1:**
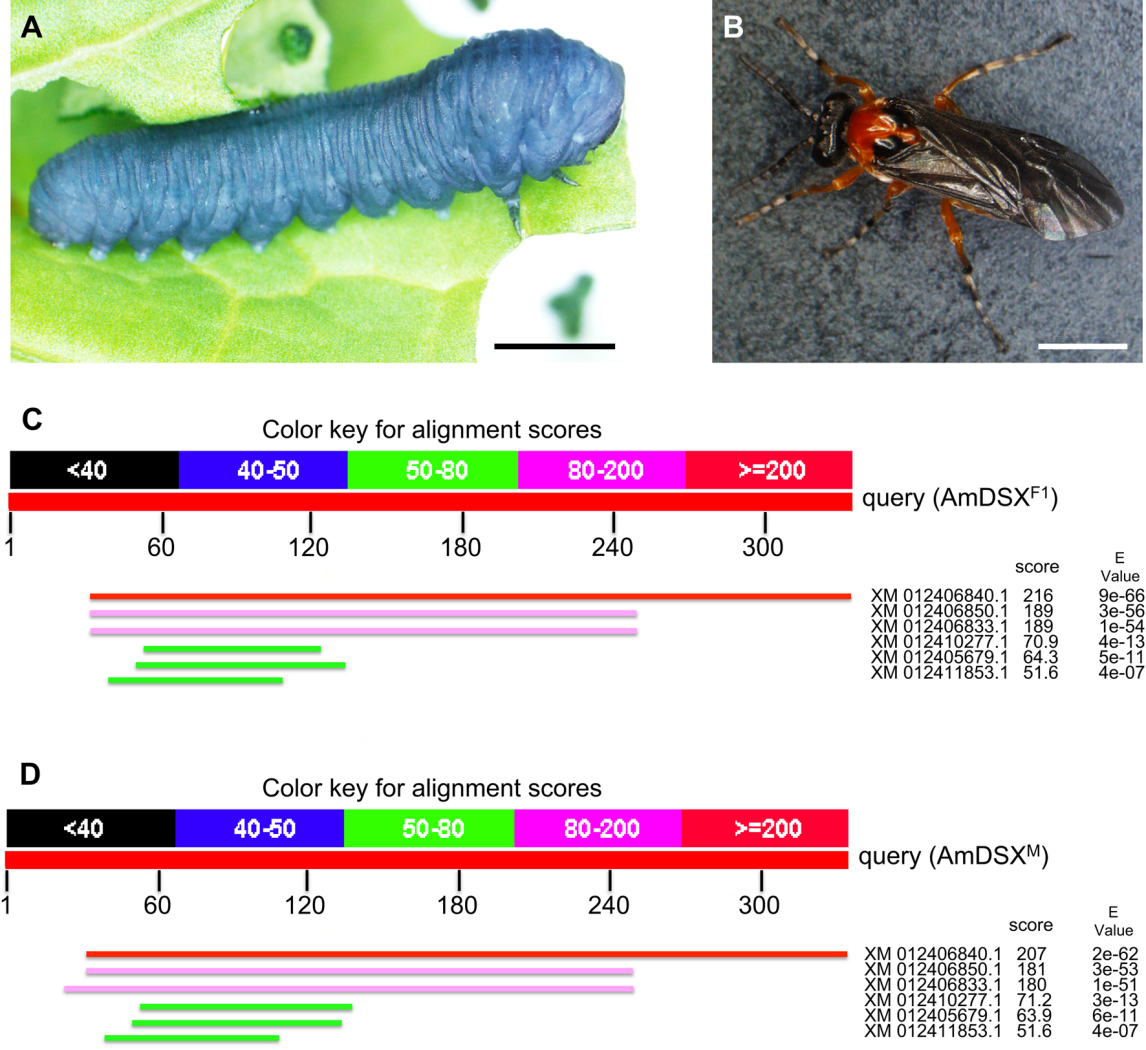
Identification of a *dsx* ortholog from *A. rosae*. Photographs of a last-instar larva (**a**) and adult male (**b**) of *Athalia rosae*. *Scale bars* indicate 2 mm. A tblastn search of the NCBI database was performed, specifying an *A*. *rosae* dataset, using the full amino acid sequence of DSX in *Apis mellifera* (AmDSX) as a query sequence. Distribution of six blast hits on the query sequence is described. One predicted gene (NCBI accession number XM_012406840.1) showed significant similarities to female-specific AmDSX isoform, AmDSX^F1^ (**c**) and male-specific AmDSX isoform, AmDSX^M^ (**d**)

To verify this hypothesis, it is quite important to know whether hymenopteran species, which are more primitive than *Aculeata* species, also have *csd* and *fem* orthologs. The sawfly, *Athalia rosae* (Hymenoptera: Tenthredinidae), belongs to the *Symphyta* infra-order, which is the most primitive infra-order in Hymenoptera (Fig. 1a, b). Adults of this species show significant sexual dimorphisms in their gonads and genitalia (Hatakeyama et al. 1990a, b; Oishi et al. 1995). The male genitalia form a well-organized capsule, in repose retracted within the apical segments of the abdomen, as those reported in other sawfly species (Schulmeister 2003). In particular, the genitalia of females consist of a unique ovipository apparatus with a saw tooth-like structure, which is characteristic for the sawfly species (Ross 1945). Classical genetic analysis demonstrates that sexual fate in this species is also determined by the single-locus CSD system (Naito and Suzuki 1991). The number of alleles at this locus in a field population calculated by random crossing is 40–50 (Fujiwara et al. 2004). However, to date, genes involved in sex determination and sexual differentiation have not been identified in this species.

The whole genome sequencing (WGS) and assembly of *Athalia rosae* were conducted by the i5K Initiative (Baylor College of Medicine, https://www.hgsc.bcm.edu/arthropods/turnip-sawfly-genome-project) and the assembled data was submitted to the NCBI database in 2013 (GenBank assembly accession number GCA_000344095.1 Aros_1.0). Sequencing depth and coverage are highly sufficient (genome coverage 467.2×), and statistics of the assembly (number of scaffolds 522; number of contigs 7588; contig N50, 51,418; contig L50, 825) shows that the quality of the *Athalia rosae* genome assembly is good enough for sequence analyses. By using this *Athalia rosae* WGS data, here, we attempted to identify *csd*, *fem*, and *dsx* orthologs from the *A*. *rosae* genome to gain insights into whether the molecular mechanism for the sex determination observed in *Aculeata* species is conserved in Symphytan species. As a result, we successfully identified a sawfly ortholog of *dsx*, but failed to find genes homologous to *csd* and *fem* in the honeybee, *Apis mellifera*. The *dsx* ortholog was designated as *Ardsx* and its functions in sexual differentiation were assayed by RNAi analysis. Here we provide several lines of evidence that *Ardsx* is necessary for male development in the sawfly.

## Materials and methods

### Insect

The wild-type sawflies (*Athalia rosae*) and several mutant strains *cream eye color* (*cec*), *short wing* (*sw*), and *yellow fat body* (*yfb*), which had been kept at the National Agriculture and Food Research Organization, were used in this study. General biology of *A. rosae* is described in Oishi et al. (1993). Animals were reared continuously at 25 °C under 16 h light, 8 h dark conditions. The eggs were stored in plastic containers with sufficient humidity, and we used Japanese radish leaves (Sakata no tane) for larval feed. A hydroponic culture kit (Green Farm) was used for cultivation of the Japanese radish leaves. The *cec* gene is involved in pigmentation of eye, and animals homozygous for *cec* display cream-eye color (Lee et al. 1998). Adult wings of animals homozygous for *sw* become atrophied. Animals homozygous for *yfb* have fat bodies with yellow color (Sawa and Oishi 1989). On the basis of the phenotype of the recessive inheritance trait described above, we discriminated diploid females from fertilized eggs from haploid males from unfertilized eggs.

### RNA extraction and RT-PCR

Extraction of total RNA was performed using ISOGEN (Nippon Gene, Tokyo, Japan) according to the protocol provided by the manufacture. A homogenization pestle (Funakoshi) was used for homogenizing samples. Total RNA extracted from samples during embryonic stage to young larval stages (2 days after hatching) were precipitated by addition of 1 μl of glycogen (20 mg/mL, Wako Junyaku) per sample. RT-PCR reactions were performed according to the protocol described previously (Suzuki et al. 2012). The ArEF1-LP and ArEF1-RP primers were used to amplify *A. rosae* elongation factor-1 alpha (*EF*-*1*) (NCBI accession number AB253792) as a positive control for the RT-PCR reaction. The primer sequences utilized in this study are indicated in Table 1. PCR products were analyzed on a 2% agarose gel and visualized with ethidium bromide.

**Table 1:** Primer sequences and PCR conditions utilized in this study

Target gene	Primers	Sequence (5′ → 3′)
*Ardsx*	ArdsxFMF1	AAAGATGCACAAGCCGATTTG
	ArdsxFMR1	GGCTGATGAACAAGGCTCATC
	Ardsx1	CATAATGGATCAAAACGACAAC
	Ardsx2	GACGTGATTATCCCGATAAAT
	Ardsx7	TTATCCCGATAAATCGTATAAATCTTC
*EF*-*1*	ArEF1-LP	CTTCACTCTTGGTGTCAAGCAGCTC
	ArEF1-RP	ACATCCTGAAGAGGAAGACGGAGAG

### Quantitative real-time RT-PCR

qRT-PCR assays were performed according to the protocol described previously (Suzuki et al. 2012). The ArEF1-LP and ArEF1-RP primers were used to amplify elongation factor-1 alpha (*EF*-*1)* as an internal standard for quantification. All primer sequences used in the qRT-PCR assays are listed in Table 2.

**Table 2:** Sequences of primers used for qRT-PCR

Target gene	Primers	Sequence (5′ → 3′)
*Ardsx*	Ardsx real-time F1	GCGGGTCAAATGGATTCCAAC
	Ardsx real-time R1	GCCCTTCTGAGTGCAGTTTGC
*EF*-*1*	ArEF1-LP	CTTCACTCTTGGTGTCAAGCAGCTC
	ArEF1-RP	ACATCCTGAAGAGGAAGACGGAGAG

### Rapid amplification of cDNA ends (RACE)

RACE was performed on the basis of previous protocols (Suzuki et al. 2010), except that the cDNA templates were prepared from whole bodies of pupae. All primer sequences used in RACE are listed in Table 3.

**Table 3:** Sequences of primers used for RACE

Target gene	Primers	Sequence (5′ → 3′)
*Ardsx*	ArdsxRACEF1	ACTGCAACTGCTATTCGCCGTATCG
	ArdsxRACER1	CCGAACGTGGGTCCACCTGTTCTTC
	ArdsxRACEF2	CGCAGTGATATGTGCAGCAGAATCG
	ArdsxRACER2	GCAGTTTGCAGCGCCATAACTCTTTG
	ArdsxRACEF3	GCGCCATTTTGGCCTGTCGATAATAC
	ArdsxRACER3	CAACGCGCACAATTCGGTGGTGTAC
	Ardsx4-2F1	CGTCGCGCGTTTTCAAAAGAC
	Ardsx3-2F1	GCTGTGTGGTCGTCGTTCAAG
	Ardsx3-3F1	ACACTCTGCCGCATGCATTTC
	Ardsx3-1F1	GAGTTACCGACTGCATGGAAG
	Ardsx4-1F1	AGTTTGATACGGCTACCGTTC
	Ardsx3-5F1	TTGGTGGCAATTGACTGAAAG
	Ardsx3-6F1	CGCGCATCGGAGGTTCGCAAC

### Preparation of dsRNAs

Two sequences conserved between the *Ardsx* isoforms were amplified with primer pairs ArdsxdsRNAF1-ArdsxdsRNAR1 and ArdsxdsRNAF2-ArdsxdsRNAR2 (Table 4), and they served as a DNA template for double-stranded RNA (dsRNA) synthesis. Each primer contained a T7 promoter site. The dsRNA synthesis was performed according to the protocol described previously (Suzuki et al. 2012).

**Table 4:** Sequences of primers used for synthesis of dsRNA

Target gene	Primers	Sequence (5′ → 3′)
*Ardsx*	ArdsxdsRNAF1	CCGGATCCTAATACGACTCACTATAGGGCGGAAGAACAGGTGGACCCAC
	ArdsxdsRNAR1	CCGGATCCTAATACGACTCACTATAGGGCGCAATTCGTCGAGATGCTTC
	ArdsxdsRNAF2	CCGGATCCTAATACGACTCACTATAGGGCGCTGACTTCACTGCTGCAGCGC
	ArdsxdsRNAR2	CCGGATCCTAATACGACTCACTATAGGGCGCAACGGAGCTAATCTCTGAAC

### Injection of dsRNAs into insects

The larvae and pupae were anesthetized by chilling for 30 min in a plastic container placed in an ice bath (Yoshiyama et al. 2013). Individuals receiving injection were left on an ice pack during the injection procedure. Injection was performed using a handmade injection apparatus (Hatakeyama et al. 1990a, b): a fine glass needle, made by pulling a 25-μl Microcaps (Drummond) and cutting the tip, was connected to a 1-ml plastic syringe. dsRNA was injected into the dorsal hemocoel in the second abdominal segment of the larva. Synthesized dsRNA was adjusted to a final concentration of 1 mg/ml, and injected at 3 mg per individual.

### Phylogenetic analysis of *Ardsx*

Multiple alignment analysis of the complete amino acid sequences of *Ardsx* and known *doublesex* orthologs was performed using Clustal X 2.0.11 (http://www.softpedia.com/get/Science-CAD/Clustal-X.shtml) with the following settings (pairwise alignment parameters: gap opening penalty 17.00, gap extension penalty 0.2, identity protein weight matrix; multiple alignment parameters: gap opening penalty 17.00, gap extension penalty 0.2, delay divergent cutoff 30%, identity protein weight matrix). Matrices of amino acid *p* distances were calculated using the program MEGA version 4.0. A comparative phylogenetic tree was produced using the amino acid *p* distance method and the neighbor-joining algorithm with a bootstrap value of 1000.

### Observation of internal and external genital organs

When preparing cuticle specimens of external genitalia, dissection was performed in 70% ethanol. The genitalia were trimmed and then dehydrated in 100% ethanol. The trimmed genitalia were put in a mixed solution of Canada balsam and methyl salicylate on a microscope slide, and covered with a glass coverslip to make cuticle specimens. The specimens were kept at 60 °C for 24 h, and observed under a stereoscope (Olympus SZX 7). To capture images, a CCD camera (Olympus DP 7) mounted on the stereoscope was used. The images were analyzed with CellSens standard software (Olympus). Adult gonads and internal reproductive organs were dissected out immediately after emergence in 1 × PBS. The dissected organs were observed using the stereoscope as described above.

## Results

### Homology-based search of sex-determining genes from *Athalia rosae* genome

Genetic analysis demonstrates that sexual fate in *A. rosae* is determined by the single-locus CSD system (Naito and Suzuki 1991). To investigate whether the molecular basis for the sex-determining mechanism of this insect is the same as those reported in honeybee species, a tblastn search of the NCBI database was performed, specifying an *A*. *rosae* dataset containing 22,130 of gene models, using the full amino acid sequence of CSD, FEM, and DSX in *Apis mellifera* as a query sequence. As a result, we were not able to find any sequences with significant homology to CSD and FEM. On the other hand, the tblastn search retrieved one predicted gene (NCBI accession number XM_012406840.1) with significant higher similarity (hit score >200, *E* value <e−60), when either female (AmDSX^F1^) or male-specific isoform (AmDSX^M^) of *Apis mellifera* DSX served as a query sequence (Fig. 1c, d). Comparison of the predicted protein encoded by the retrieved gene with proteins in the NCBI database using the blastp program indicated that XM_012406840.1 encodes a protein showing high homology to the female-specific DSX isoform (AmDSX^F2^) of *A. mellifera* with 54% identity and 69% similarity and also to the female-specific DSX isoform (TcDSX^F3^) of *Tribolium castaneum* with 49% identity and 59% similarity. Prediction of conserved protein domains using Pfam demonstrated that the protein encoded by XM_012406840.1 has a DM domain characteristic of the DM superfamily genes containing *dsx*, *mab3*, and *Dmrt* and the oligomerization domain that is characteristic for insect DSX. Taken together, we concluded that the predicted gene (XM_012406840.1) is an *A. rosae* ortholog of *dsx*, and hereafter we label this gene “*Ardsx*”.

### Molecular cloning of full-length *Ardsx* cDNAs from males and females

**Fig. 2:**
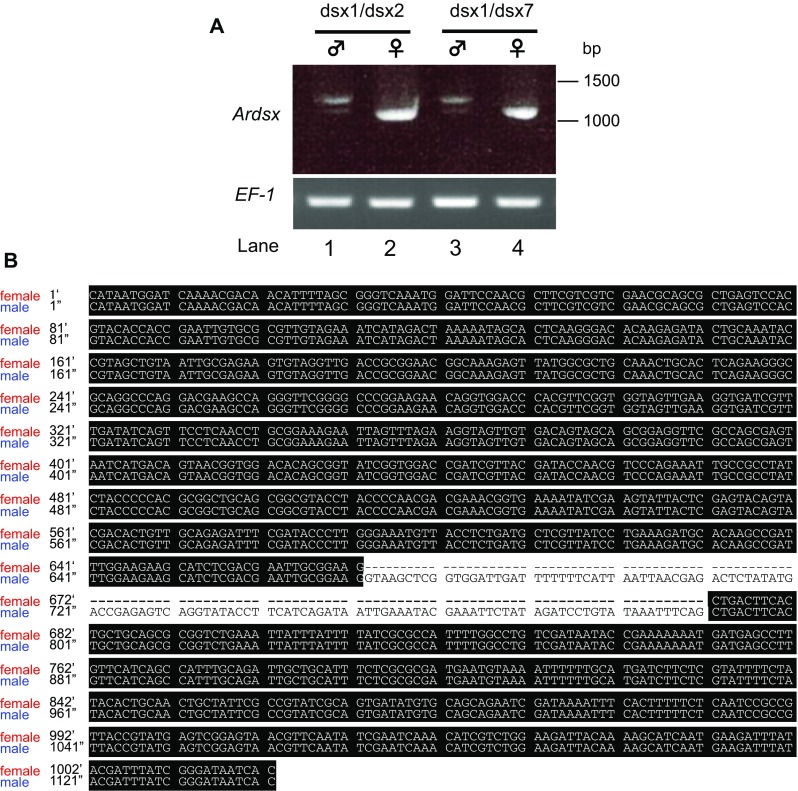
RT-PCR analysis of *Ardsx* expression in male and female pupae. **a** RT-PCR for expression pattern analysis of *Ardsx* mRNAs with primers that were designed on the basis of the nucleotide sequence of the predicted gene (XM_012406840.1) using cDNAs prepared from male and female pupae. Expected size of amplified product using a primer pair dsx1 and dsx2 is 1025 bp, whereas a primer pair dsx1 and dsx7 is expected to amplify 1018 bp of the DNA fragment. The approximate location of the primers (Ardsx1, Ardsx2, and Ardsx3) are described by *red arrows* in Fig. 3a. The *upper panel* shows *Ardsx* expression. The *bottom panel* shows amplification of the *Ar EF*-*1* alpha (*EF*-*1*) transcript, which served as a positive control for RNA extraction and RT-PCR. PCR products were separated on 2% agarose gels and stained with ethidium bromide. Molecular sizes, presented in base pairs, are indicated to the* right* of each panel. **b** Comparison of nucleotide sequences of RT-PCR products amplified from male and female larvae. *Dashes* show alignment gaps. Nucleotides identical between both sexes are shaded* black*. Numbering is relative to the 5′ end of each of the RT-PCR product

To examine whether the aforementioned genes retrieved by the tblastn searches are transcribed in vivo, we first performed RT-PCR analysis with primers that were designed on the basis of the nucleotide sequence of the predicted gene using cDNAs prepared from male and female pupae. The RT-PCR with primers dsx1 and dsx2 (amplicon size 1025 bp) amplified a DNA fragment whose size was almost similar to the predicted sizes in females (Fig. 2a, lane 2). On the other hand, in males, the same PCR resulted in an amplified product of approximately 1200 bp, which was larger than the expected size (Fig. 2a, lane 1). Similar results were obtained when the RT-PCR was performed with primers dsx1 and dsx7, which were designed to amplify a 1018-bp cDNA fragment (Fig. 2a, lanes 3 and 4). These RT-PCR products were cloned and sequenced. The sequences of the DNA fragments amplified from females were identical to that of XM_012406840.1, while the PCR products obtained from males were found to contain an insertion of 119 bp sequence (Fig. 2b). A blastn search of the *A*. *rosae* genome sequence (Version GCA_000344095.1 Aros_1.0) revealed that scaffold 11 contained the region encoding the whole sequence of the aforementioned cDNAs, which spanned at least 15 kb. Comparison between the cDNAs and the genomic DNA sequence demonstrated that the 119-bp fragment was derived from a single intronic sequence. Thus we concluded that inclusion of this intron in the pre-mRNA processing yields male-specific products. This strongly suggests that *Ardsx* in the sawfly may be sex-differentially spliced like *dsx* orthologs identified in other insects.

**Fig. 3:**
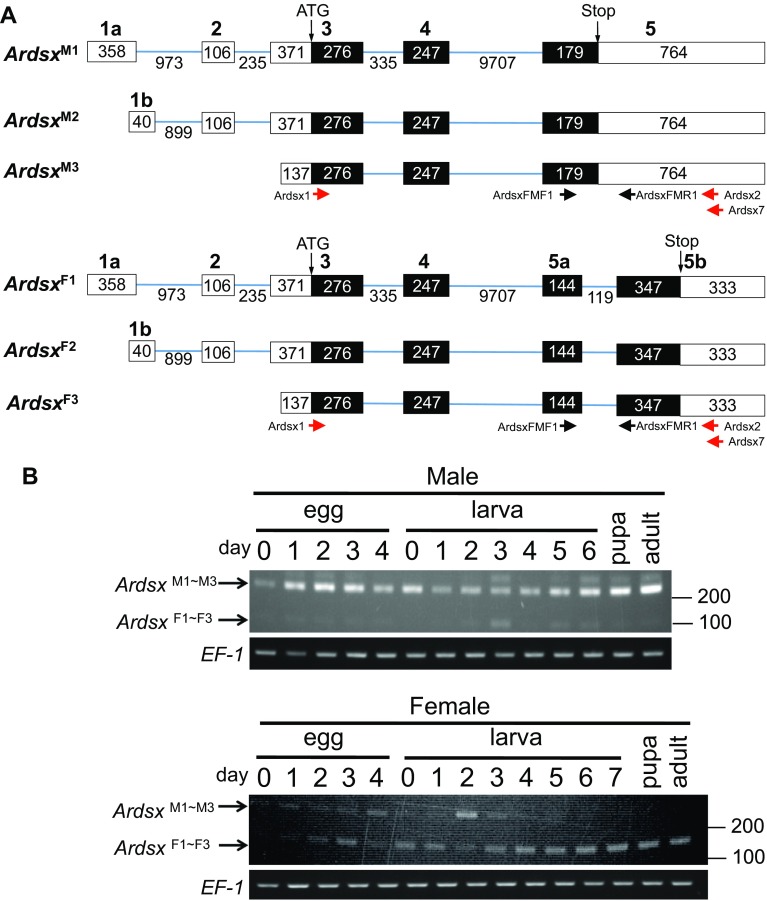
Structures of the *Ardsx* splicing variants obtained by RACE and sexual difference in the expression pattern of *Ardsx*. **a** *Upper panel* shows the three male-specific *Ardsx* transcription variants (*Ardsx* ^M1^, *Ardsx* ^M2^, and *Ardsx* ^M3^) and* lower panel* indicates three female-specific *Ardsx* transcription variants (*Ardsx* ^F1^, *Ardsx* ^F2^, and *Ardsx* ^F3^) obtained by RACE. *Boxes* represent exons and *lines* are introns. Only exons are drawn to scale. The *white regions* indicate UTRs. The *black regions* indicate ORFs. The *numbers* shown above the *box* are exon numbers. The *number* indicated in each region describes the size in base pairs. ATG sites and stop codons are indicated. The *red arrows* show the approximate position of the primers (Ardsx1, Ardsx2, and Ardsx3) used for RT-PCR analysis illustrated in Fig. 2a. The *black arrows* indicate the approximate location of the primers (ArdsxFMF1 and ArdsxFMR1) that were used for RT-PCR described in **b**. **b** Sexual difference in the expression pattern of *Ardsx* mRNA was assessed by RT-PCR with primers described in **a**. Individuals hatched from fertilized eggs served as females, and individuals hatched from parthenogenetic eggs served as males. For embryonic stages, total RNA was extracted from a single egg at day 0, day 1, day 2, day 3, and day 4 after oviposition or parthenogenetic treatment. For larval stages, total RNA was extracted from a single animal at day 0–day 6 after hatching for males, and at day 0–day 7 after hatching for females since the larval period of females was 1 day longer than that of males. Total RNA was also isolated from a pupa at day 7 after pupation and an adult at day 5 after emerging. *Bottom panel* shows the results of RT-PCR amplification of the *EF*-*1* transcript, which served as a positive control for the RT-PCR reaction. PCR products were analyzed on a 2% agarose gel. *Arrows* to the* left* of the gel refer to position of female and male specific-isoforms of *Ardsx* (*Ardsx* ^F1–F3^ and *Ardsx* ^M1–M3^). Molecular sizes, presented in base pairs, are indicated to the* right* of each panel

Next, we carried out RACE using total RNA samples purified from whole bodies of female and male pupae to determine the full-length coding and 5′ and 3′ sequences of the *Ardsx* gene. The 5′ ends of the *Ardsx* cDNAs determined by RACE were consistent with the 5′ end of the predicted gene XM_012406833.1. In addition, our 5′RACE amplified another two cDNA fragments, which respectively started at 698 and 380 nt upstream of the 5′ end of the predicted gene XM_012406833.1. Because no open reading frame of significant length was identified in these new fragments, we regarded them as 5′ UTRs. The 3′ RACE resulted in a single amplified product with a nucleotide sequence identical to the XM_012406840.1. The nucleotide sequences obtained by the aforementioned RACE showed no difference between males and females. Sex-specific difference was restricted to either presence or absence of the 119-bp sequence described in Fig. 2b. Consequently, *Ardsx* appeared to yield three male-specific variants (*Ardsx* ^M1^, *Ardsx* ^M2^, and *Ardsx* ^M3^) and three female-specific variants (*Ardsx* ^F1^, *Ardsx* ^F2^, and *Ardsx* ^F3^) (Fig. 3a). The difference among these variants rests entirely in the 5′ UTR, owing to alternative transcription start sites. Comparison between the full-length *Ardsx* cDNAs and the genomic sequence revealed that exon 5 was differentially spliced between male and female-specific isoforms (Fig. 3a). In the female isoforms, the male-specific 119-bp sequence, which contains a stop codon, was spliced out, causing amino acid sequence difference in the C-terminal region between male and female ArDSX isoforms.

**Fig. 4:**
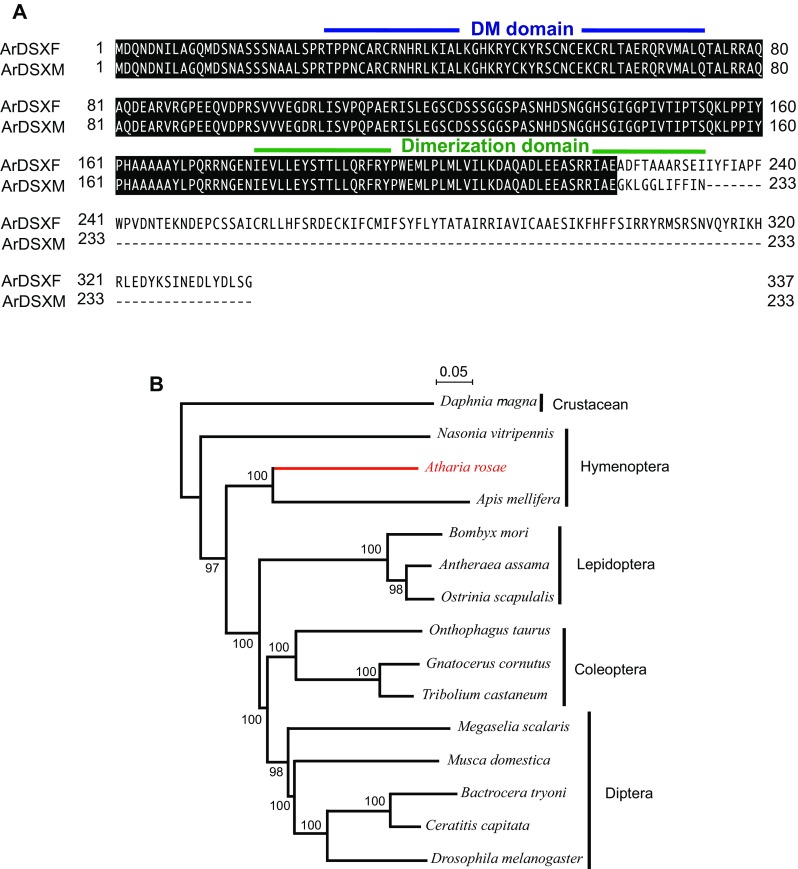
Amino acid sequence of ArDSX and phylogeny of ArDSX with other insect DSX proteins. **a** Amino acid sequence comparison between ArDSX^F^ and ArDSX^M^.* Dashes* show alignment gaps. Amino acids identical between both proteins are shaded* black*. The* blue* and the* green lines* indicate DM domain and dimerization domain, respectively. **b** A comparative phylogenetic tree was produced using the complete amino acid sequences of ArDSX^F^ and 14 other known proteins, including DSX beta protein from *Daphnia magna* (BAJ78308), DSX^F1^ from *Apis mellifera* (ABW99105), BmDSXF from *Bombyx mori* (NP_001036871), DSX^F1^ from *Onthophagus taurus* (AEX92939), DSX^F^ proteins from *Nasonia vitripennis* (NP_001155990), *Ostrinia scapulalis* (BAJ25852), *Antheraea assama* (ADL40852), *Gnatocerus cornutus* (BAW32685), *Tribolium castaneum* (AFQ62106), *Megaselia scalaris* (AAK38831), *Musuca domestica* (AAR23812), *Bactrocera tryoni* (AAB99948), *Ceratitis capitata* (AAN63598), and *Drosophila melanogaster* (NP_001287220). Bootstrap values for 1000 replicate analyses are shown at the branching points. The *bar* below the tree indicates the branch length representing the mean number of differences (0.05) per residue along each branch

*Ardsx* ^M1^, *Ardsx* ^M2^, and *Ardsx* ^M3^ encode the same protein (ArDSX^M^) of 233 amino acids. *Ardsx* ^F1^, *Ardsx* ^F2^, and *Ardsx* ^F3^ also encode the protein with an identical sequence of 337 amino acids (ArDSX^F^). ArDSX^M^ and ArDSX^F^ share an N-terminal region of 222 amino acids that encodes a conserved DNA-binding domain called DM domain (or OD1 domain) and part of a conserved dimeriztion domain known as OD2 domain, which is specifically conserved among DSX proteins (Price et al. 2015) (Fig. 4a). Amino acid sequence of the C-terminal part shows difference between ArDSX^M^ and ArDSX^F^ (Fig. 4a).

Phylogenetic analysis using the full amino acid sequence of ArDSX^F^ indicated that the identified sequence from *A. rosae* was grouped with other hymenopteran DSX proteins (Fig. 4b). These results strongly suggest that *Ardsx* is a *dsx* ortholog of *A. rosae*.

### Expression analysis of *Ardsx*

As shown above, exon 5 of *Ardsx* was differentially spliced between two sexes, resulting in the male-specific insertion of the 119-bp fragment (Fig. 3a). To investigate whether sexual difference in the expression pattern of *Ardsx* mRNA changes according to the developmental stages, RT-PCR analysis was performed using cDNAs prepared from males and females at the different stages with primers that specifically anneal to the regions flanking the 119-bp fragment (Fig. 3b).

As a result, the amplification product of 269 bp was observed in males at all the examined stages (Fig. 3b). On the other hand, the same RT-PCR amplified the 150-bp DNA fragment in females throughout the examined stages. In females, the 269-bp DNA fragment was weakly detectable until 4 days after hatching. After that, the DNA band gradually disappeared with a concomitant increase in the amplification level of the 150-bp DNA fragment. After cloning of these amplified products and sequencing the cloned DNA, the amplified product of 269 bp corresponded to the male-specific *Ardsx* isoforms identified by RACE, while the RT-PCR product of 150 bp had a nucleotide sequence identical to that of the corresponding region in the female-specific *Ardsx* isoforms. These results clearly demonstrate that *Ardsx* pre-mRNA is spliced alternatively in a sex-dependent manner. However, in females, sex-specificity of the splicing is less accurate until middle larval stages, yielding the male-specific *Ardsx* in addition to the female-specific *Ardsx* isoforms.

### Functional analysis of *Ardsx*

**Fig. 5:**
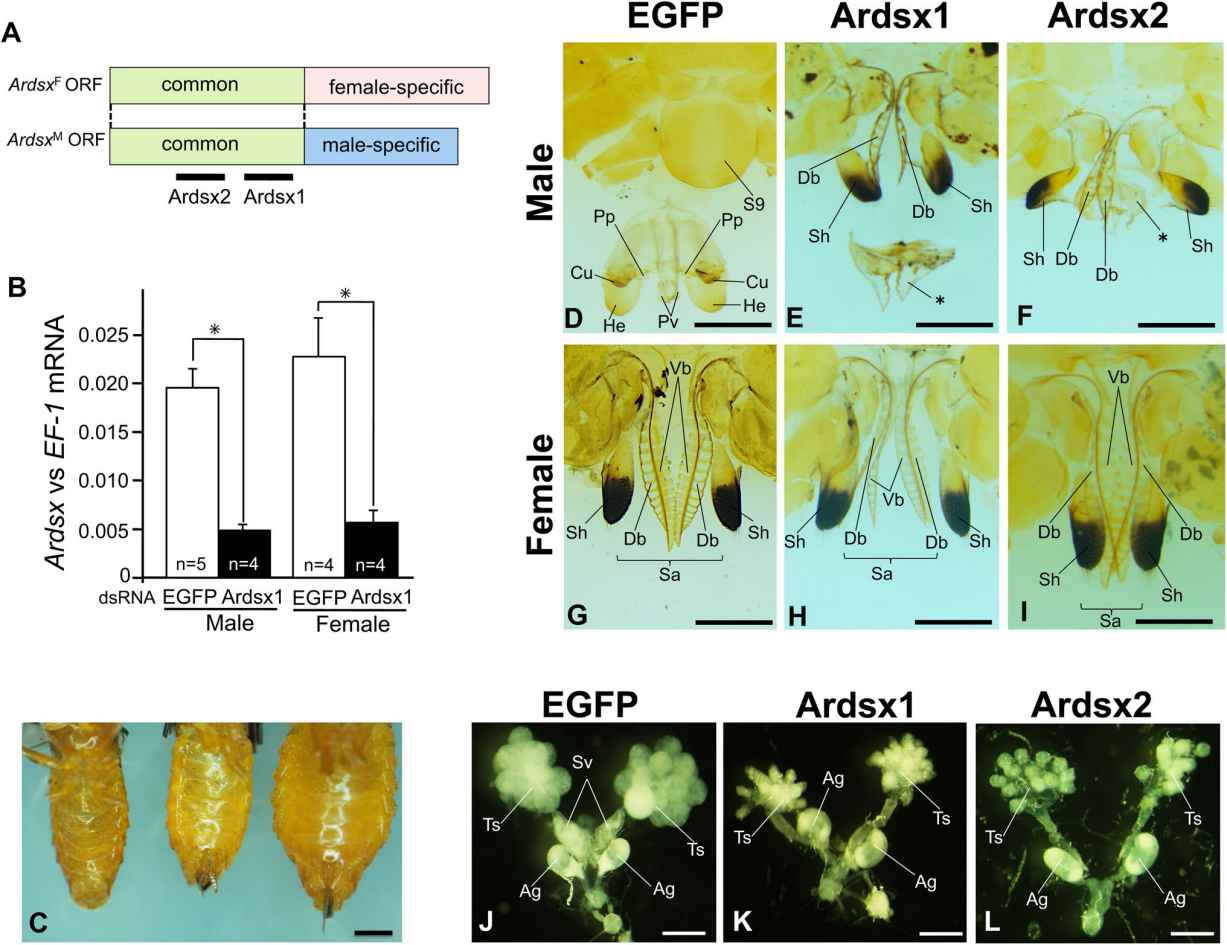
Effects of RNAi-mediated knockdown of *Ardsx* on sexual development. **a** Schematic diagram of two dsRNAs (Ardsx1 and Ardsx2) targeting *Ardsx* mRNA. **b** Quantification of *Ardsx* mRNA expression 1 day after injection by qRT-PCR. *EF*-*1* served as an internal standard. *Error bar* standard deviation (SD); *significant differences at the 0.05 level (*t* test) compared with the control. **c** Ventral view of the abdominal segments of the negative control male (*left*), *Ardsx* knockdown male (*middle*), and the negative control female (*right*). Ventral view of the external genitalia in the negative control male (**d**), *Ardsx* knockdown male injected either with Ardsx1 (**e**) or Ardsx2 (**f**), the negative control female (**g**), and *Ardsx* knockdown female injected either with Ardsx1 (**h**) or Ardsx2 (**i**). *Sa* saw, *Vb* ventral pair of blades, *Db* dorsal pair of blades, *Sh* sheaths, *S9* ninth sternite, *Cu* cuspis, *He* herpe, *Pp* parapenis, *Pv* penis valve; *abnormal tissue specifically observed in *Ardsx* knockdown males. Morphologies of testes and internal reproductive organs dissected out from the control male (**j**) and *Ardsx* knockdown male injected either with Ardsx1 (**k**) or Ardsx2 (**l**). *Ts* testis, *Sv* seminal vesicle, *Ag* accessory gland

In order to analyze the function of *Ardsx*, we investigated the effects of *Ardsx* knockdown on sexual developments. Two different dsRNAs (Fig. 5a, Ardsx1 and Ardsx2), both of which targeted to a region common between *Ardsx* isoforms, were injected into larvae at the wandering stage. qRT-PCR analysis confirmed a significant reduction in *Ardsx* mRNA level in males and females injected with Ardsx1 (Fig. 5b). Similar results were observed when males and females were injected with Ardsx2 (data not shown). Morphological analysis of adult phenotypes demonstrated that negative control males and females, which were injected with dsRNA targeting the *EGFP* gene, had normal genital organs as observed in wild-type adult males and females (Fig. 5c, d, g). On the other hand, *Ardsx* knockdown males (*Ardsx* KD males) injected with Ardsx1 had external genital organs whose shapes were very similar to those observed in the control females (Fig. 5c). Normal females have external genitalia that contain an ovipository apparatus consisting of two pairs of valvifers, which give rise to the other parts: a saw formed by two pairs of blades (a ventral pair of blades derived from the first valvifers and a dorsal pair of blades derived from the second valvifers); and a sheath composed of a pair of appressed end segments of the second valvifers (Ross 1945) as represented by Fig. 5g. The genitalia observed in the *Ardsx* KD males contained several imcomplete parts of the ovipository apparatus including the dorsal pair of blades and the sheath, both of which are derived from the second valvifers (Fig. 5e). Differing from the normal female genitalia, *Ardsx* KD male genitalia lacked the ventral blades but instead contained an abnormal tissue with a saw tooth-like structure. Morphological analysis of internal reproductive organs revealed that these *Ardsx* KD males showed abnormal (Fig. 5k). Moreover, the seminal ducts looked thicker than those of the control male. The seminal vesicle filled with mature sperms in the control male (Fig. 5j) was not observed in the *Ardsx* knockdown males (Fig. 5k). Similar morphological abnormalities were observed in *Ardsx* KD males injected with Ardsx2 (Fig. 5f, l).

*Ardsx* knockdown females injected either with Ardsx1 or Ardsx2 were also subjected to the same analyses but their external genitalia showed the same phenotype as those observed in the control females (Fig. 5h, i). All the examined *Ardsx* KD females had normal ovaries and internal reproductive organs and fertile (data not shown).

These results suggest that expression of *Ardsx* during the pupal stage is essential for normal sexual development in the male external genitalia and that its expression is also important for testis and seminal vesicle development.

## Discussion

Despite the previous finding that sexual fate in *A*. *rosae* is determined by the single-locus CSD system (Naito and Suzuki 1991), we could not find genes showing significant homology to *csd* and *fem* in the *A. rosae* genome, both of which are core components for the honeybee CSD system. The *csd* was considered to have arisen from duplication of the *fem* gene (Schmieder et al. 2012) that is a *tra* homolog found in honeybees and other *Aculeata* species (Privman et al. 2013; Schmieder et al. 2012). To date, the *tra* gene has been identified not only in hymenopteran species but also in dipteran and coleopteran species. On the other hand, several previous studies and recent genome-wide analysis in a wide range of insect species revealed that *tra* shows a distinctly patchy distribution among insects (Geuverink and Beukeboom 2014). For example, no *tra* homologs have been identified in lepidopteran insects including *Bombyx mori*, *Danaus plexippus*, and *Heliconius melpomene* (Geuverink and Beukeboom 2014; Mita et al. 2004). One of the coleopteran species, *Tribolium castaneum*, carries a *tra* ortholog (Shukla and Palli 2012), while the *tra* gene has to date not been found in the genome of coleopteran *Dendroctonus ponderosae* (Geuverink and Beukeboom 2014). *Mengenilla moldrzyki* that belongs to Strepsiptera, which is a sister order of the Coleoptera, appeared to lack a *tra* homolog (Geuverink and Beukeboom 2014). The most interesting pattern of *tra* distribution is seen in the *Diptera* genus. The *tra* gene is found in brachycera species including *D. melanogaster,* while several mosquito species, which belong to a basal dipteran lineage, do not possess *tra* (Geuverink and Beukeboom 2014). These facts imply multiple independent losses or recruitment of *tra* into the sex determination cascade. It would be possible that *A*. *rosae*, which belongs to the most primitive infra-order in Hymenoptera, may not possess *tra* orthologs. It is well known that upstream components of the sex determination cascade are highly evolutionarily labile (Wilkins 1995). For example, in mosquito species, *Aedes aegypti* and *Anopheles gambiae*, maleness is determined by a dominant Y chromosome-linked *M* factor, but the gene encoding the *M* factor is completely different between these two species. *Nix*, which is a distant homolog of *tra*-*2*, functions as an *M* factor in *A. aegypti*, while *Yob*, which seemed to encode a short amino acid protein that may contain a helix-loop-helix motif, acts as an *M* factor in *A. gambiae* (Hall et al. 2015; Krzywinska et al. 2016). In the sawfly, a gene responsible for the CSD system might be different from those identified in multiple *Aculeata* species. However, we cannot rule out the possibility that our homology-based search using the tblastn program provided by NCBI may be inappropriate for identifying *tra* orthologs such as *csd* and *fem* from the *A. rosae* genome because of its less conserved sequences among groups.

In this paper, we provided several lines of evidence that a homolog of the *dsx* gene, named *Ardsx*, is also present in the *A. rosae* genome (Figs. 1c, d, 4b), and showed that *Ardsx* is transcribed into sex-specific mRNA isoforms as a result of sex-specific alternative splicing (Figs. 2, 3). The *Ardsx* mRNAs produced in males and females encode polypeptides sharing common N-terminal sequences but differing in C-terminal sequences (Fig. 4a), similar to the *dsx* orthologs identified in the honeybee and other multiple insect species. Concerning this point, it is presumed that *Ardsx* would take a splicing pattern similar to the honeybee *dsx* (*Amdsx*), but the following two points are significantly different: (1) *Ardsx* does not have a female-specific exon. In *A. mellifera*, the single female-specific exon in *Amdsx* ^F1^ is skipped in the male variant (*Amdsx* ^M^), resulting in the final two exons being shared between *Amdsx* ^F1^ and *Amdsx* ^M^ (Cho et al. 2007). Similar sex-specific splicing pattern is reported in *B. mori dsx* (Suzuki et al. 2001). In *A. rosae*, the male-specific *Ardsx* contained a stop codon located in the middle of exon 5, while the region of 119 bp including the stop codon is skipped in the female-specific *Ardsx* isoforms (Fig. 3a). In other words, *Ardsx* has a female-specific intron. (2) In all the *dsx* orthologs so far characterized, skipping of an exon including a female-specific stop codon is seen only in males, whereas in *A. rosae*, skipping of the partial exonic region that includes the male-specific stop codon occurs in females (Fig. 3a). These findings may imply that the regulatory mechanism of sex-specific splicing of *Ardsx* may be different from that of *Amdsx*.

*Ardsx* knockdown adult males had several parts of the ovipository apparatus including the dorsal pair of blades and the sheath, both of which are derived from the second valvifers (Fig. 5e, f). These results indicate that development of the dorsal pair of blades and the sheath in males is repressed by the expression of ArDSX^M^ and its expression is essential for development of the male genitalia. Differing from the normal ovipository apparatus, *Ardsx* KD male genitalia lacked the ventral pair of blades, which are derived from the first valvifers (Fig. 5e, f). In *D*. *melanogaster*, one of two isoforms of Abdominal-B (Abd-B), Abd-Bm, is present only in the female genital primordium, whereas the other isoform, Abd-Br, is present only in the male genital primordium (Casares et al. 1997; Duncan 1996). Concerted action of these two isoforms either with DSX^M^ or DSX^F^ is important for the appropriate sexual development of genital primordium (Sánchez et al. 2001). A previous study demonstrated that knockdown of *Abd*-*B* in *A. rosae* females caused a malformed ovipositor and these females could not lay eggs, while ovaries and internal reproductive organs were normal in appearance (Hatakeyama et al., unpublished data). If *Abd*-*B* in *A. rosae* also produces abdominal segment-specific isoforms like Abd-Bm and Abd-Br, then Abd-Bm in *A*. *rosae* may be able to facilitate the development of the ovipository apparatus including the dorsal pair of blades and the sheath without the help of ArDSX^F^ since these organs were observed in *Ardsx* knockdown adults with almost complete phenotypes; whereas, concerted action of ArDSX^F^ with Abd-Br may be necessary for the development of the ventral pair of blades. It is further postulated that ArDSX^M^ together with Abd-Bm represses the development of the ovipository apparatus, resulting in the formation of the ninth sternite covering the copulatory organ (“9S” in Fig. 5d), and that ArDSX^M^ plus Abd-Br promotes the development of the male copulatory organs. These hypotheses can explain the reason why the *Ardsx* knockdown males had the ovipository apparatus, which lacked the ventral pair of blades but instead had the abnormal tissue with a saw tooth-like structure. It would be expected that Abd-Br alone was not able to induce the development either of the ventral pair of blades or the copulatory organs, resulting in the formation of the abnormal tissue in *Ardsx* knockdown males. If so, then the knockdown of *Ardsx* should also cause some morphological defects in the ventral pair of blades in *Ardsx* knockdown females. However, the *Ardsx* knockdown females exhibited normal phenotype in their ovipository apparatus (Fig. 5h, i). One possible explanation for these results is that insufficient level of knockdown of endogenous *Ardsx* ^F^ expression might allow normal development of the ventral blades in the *Ardsx* knockdown females. Null mutation of *Ardsx* induced by gene targeting using TALEN and CRISPR/Cas9 will be helpful to further understand the importance of *Ardsx* functions for the development of the ovipository apparatus.

Effects of *Ardsx* knockdown on the development of internal reproductive organs seemed relatively weak. Male-to-female sex reversal such as partial ovary formation and egg production was not seen in the *Ardsx* knockdown males. *Ardsx* knockdown females were phenotypically normal and had normal fertility. In *D. melanogaster*, males homozygous for *dsx* loss-of-function mutant alleles contain gonads with spermatogenic and undifferentiated germ cells, and asexual gonads were rarely observed (Orssaud and Laugé 1982; Steinmann-Zwicky 1994). Similarly, ArDSX^M^ may be required for sperm maturation in *A. rosae*. Thus, knockdown of *Ardsx* in males caused incomplete spermatogenesis, resulting in absence of mature sperm. Taken together with the findings that in *D. melanogaster* several genes such as *fruitless* (*fru*), *intersex* (*ix*), and *hermaphrodite* (*her*) act independently or dependently to regulate some aspects of sexual differentiation, we suppose that *Ardsx* function is not only required for the proper sexual development for the internal reproductive organs but other factors also act as a downstream regulator in the sex determination cascade in *A. rosae*.

The most notable result in this study is that knockdown of *Ardsx* caused ectopic formation of the ovipository apparatus containing the dorsal pair of blades and the sheath in males, both of which showed almost complete phenotype (Fig. 5e, f). This finding would seem to imply that formation of several parts of the ovipository apparatus represent the default state and do not require the expression of *Ardsx*. Hymenoptera is the most basal lineage in the phylogeny of holometabolous insects (superorder Endopterygota), being an outgroup to Diptera, Lepidoptera, and Coleoptera (Savard et al. 2006; Trautwein et al. 2012; Misof et al. 2014; Peters et al. 2014). The *dsx* gene in *Daphnia magna* that belongs to cladocera species, which are the closest relatives to the insects, is expressed predominantly in males and only required for male development (Kato et al. 2011). These findings lead to the inference that female sexual development might occur by default in ancestral insect species. To examine whether this hypothesis is correct or not, it will be important to reveal functions of *dsx* orthologs in other hymenopteran species.
